# Novel kinase-activating genetic events in non-small cell lung carcinomas

**DOI:** 10.37349/etat.2025.1002330

**Published:** 2025-07-09

**Authors:** Elena V. Preobrazhenskaya, Rimma S. Mulkidjan, Fyodor A. Zagrebin, Alexandr A. Romanko, Evgeniya S. Saitova, Polina R. Korzun, Jeyla O. Binnatova, Vladislav I. Tiurin, Ilya V. Bizin, Evgeny N. Imyanitov

**Affiliations:** IRCCS Istituto Romagnolo per lo Studio dei Tumori (IRST) “Dino Amadori”, Italy; ^1^Department of Tumor Growth Biology, N.N. Petrov Institute of Oncology, 197758 St.-Petersburg, Russia; ^2^Department of Medical Genetics, St.-Petersburg State Pediatric Medical University, 194100 St.-Petersburg, Russia

**Keywords:** Lung cancer, kinases, fusion, mutation, expression, RNA, next generation sequencing (NGS)

## Abstract

**Aim:**

This study aimed at the identification of new druggable alterations in non-small cell lung carcinomas (NSCLCs).

**Methods:**

RNA next generation sequencing (NGS) analysis for 650 protein kinase genes was performed for 89 NSCLCs obtained from young-onset and/or female non-smokers, who were negative for activating events involving *EGFR*, *ALK*, *ROS1*, *RET*, *MET*, *NTRK1/2/3*, *BRAF*, *HER2*, *KRAS*, or *NRAS* genes.

**Results:**

RNA sequencing identified 32 in-frame rearrangements, including 9 instances of fully preserved and 8 tumors with partially preserved tyrosine kinase domains. These 17 translocations were further analyzed in 1,059 mutation-negative NSCLCs, which resulted in the identification of two additional tumors with *ADK*::*KAT6B* rearrangement and one carcinoma carrying *RPS6KB1*::*VMP1* fusion. The recently reported *CLIP1*::*LTK* gene fusion was tested in 2,754 NSCLCs, which were negative for all known actionable mutations, however, no new instances of this translocation have been observed. We further analyzed RNA sequencing results of 89 NSCLCs for mutations affecting the kinase domain of the involved gene. There were 53 substitutions with a combined annotation dependent depletion (CADD) score above 25; all these lesions turned out to be unique, as the analysis of 551 additional NSCLCs revealed no recurrent alterations. *ROS1*, *LTK*, and *FGFR4* high-level overexpression was observed in 1 out of 89 tumors each.

**Conclusions:**

This study demonstrates the scarcity of yet unknown kinase-activating alterations in NSCLCs.

## Introduction

The majority of non-small cell lung carcinomas (NSCLCs) are driven by activation of the MAPK pathway. This upregulation occurs due to genetic alterations affecting transmembrane tyrosine kinase receptors or downstream members of this signaling cascade. EGFR and ERBB2 (HER2) receptors are usually activated via point mutations, small in-frame deletions, or insertions [1, 2]. *KRAS*, *NRAS*, and *BRAF* genes drive carcinogenesis via nucleotide substitutions affecting hot-spot codons [3, 4]. ALK, ROS1, RET, NTRK1, NTRK2, and NTRK3 receptor tyrosine kinases are activated via gene rearrangements [5–7]. *MET* gene often undergoes mutations affecting splice site and resulting in exon 14 skipping and, consequently, dramatic elevation of the stability of corresponding protein [8]. In addition, *MET* and *ERBB2* (*HER2*) overexpression due to amplification contributes to the development of some lung malignancies, although the role of these events is less strictly established as compared to gene mutations or rearrangements [9–11]. The development of EGFR, ERBB2 (HER2), ALK, ROS1, RET, NTRK1/2/3, and KRAS G12C inhibitors has changed the landscape of systemic NSCLC treatment, with approximately 2–3-fold improvement of life expectancy in patients with oncogenic mutations and 5–7-fold increase of overall survival in subjects with druggable kinase fusions [12].

All the driver events described above are mutually exclusive. Furthermore, with the exception of *KRAS*, druggable genetic alterations are enriched in NSCLCs affecting females and non-smokers. *ALK*, *ROS1*, *RET*, and *NTRK1/2/3* rearrangements demonstrate a significant association with age, being overrepresented in young-onset patients. In fact, over 70–90% NSCLCs arising in female non-smokers and/or young subjects carry an activating event in one of the above genes [12]. Consequently, the genomic analysis of patients who belong to the above categories and lack alterations in *EGFR*, *ERBB2* (*HER2*), *ALK*, *ROS1*, *RET*, *NTRK1/2/3*, *KRAS*, or *NRAS* genes has significant potential for identifying new drivers of lung cancer pathogenesis. This study focused on potentially activating events in kinase genes, given that kinases are generally accessible for therapeutic targeting.

## Materials and methods

### NSCLC patients for kinome sequencing

NSCLC tumors were collected from diagnostic samples, which were forwarded to the N.N. Petrov Institute of Oncology (St.-Petersburg, Russia) between 2020–2022. The study included formalin-fixed paraffin-embedded (FFPE) specimens obtained from non-smokers with mutation-negative NSCLC, of whom 76 patients were younger than 50 years and 13 subjects were non-smoking women aged 58–79 years. The proportion of tumor cells in the analyzed FFPE sections was above 70%.

### RNA-based next generation sequencing

RNA was extracted from manually dissected tumor cells with the PureLink FFPE Total RNA Isolation Kit (Thermo Fisher Scientific) according to the manufacturer’s protocols. RNA was purified with DNase I (Thermo Fisher Scientific) and quantified with the Qubit RNA BR Assay Kit (Thermo Fisher Scientific). RNA samples were subjected to library preparation with the KAPA RNA HyperPrep kit (Roche Sequencing Solutions, Inc.). Target enrichment was performed using the HyperCap Target Enrichment kit (Roche Sequencing Solutions, Inc.) and custom probe panels covering coding sequences of 650 kinase-related genes in accordance with the manufacturer’s guidelines. Kinase genes were divided into two groups according to the basic level of expression in lung tissues [see The HUMAN PROTEIN ATLAS resource (https://www.proteinatlas.org)]: a panel of 207 genes with a high level of expression and a panel of 443 genes with low and medium levels of expression (Tables S1 and S2). The original panels were developed by the HyperDesign Tool (Roche Sequencing Solutions, Inc.) with the specified stringency (maximum close matches: 5–10; overhang: 30 bp). Sequencing was performed on the NextSeq 2000 System (Illumina, USA) in a paired-end mode for 150 cycles in both orientations.

### Bioinformatic analysis

NGS (next generation sequencing) data were analyzed by the STAR-Fusion bioinformatic pipeline (V.1.4.0) for the identification of gene fusions and the MuTect2 tool (GATK 4.3.0.0) for the analysis of somatic mutations [13, 14]. All alterations were manually curated using the IGV (https://igv.org) and the Golden Helix GenomeBrowse tool (https://www.goldenhelix.com/products/GenomeBrowse).

The analysis of gene expression was performed for BAM-files after STAR alignment with the RSEM instrument [15]. The threshold for overexpression was a 100-fold elevation for 207 genes with a high level of expression and a 250-fold increase for low/medium expressors, with 75% quartile taken as a base-line.

### Extended NSCLC study

The analysis of frequencies of newly identified fusions was performed by variant-specific PCR. Newly identified mutations were analyzed by allele-specific PCR. Primers and probes are presented in Tables S3 and S4. PCR reactions were performed on the CFX-96 Real-Time PCR Detection System (Bio-Rad, USA). PCR mix contained 1 μL of cDNA sample, 1× GeneAmp PCR Buffer I (Applied Biosystems, USA), 250 μM of each dNTP, 200 nM of each primer and probe, 2.5 mM MgCl_2_, and 1 U of TaqM polymerase (AlkorBio, Russia) in a total volume of 20 μL. PCR reactions were initiated by the enzyme activation (95˚C, 10 min.) and included 38 cycles (95˚C for 15 s followed by 58˚C for 1 min.). Gene fusions were analyzed in 1,059 NSCLC samples negative for common druggable mutations. The analysis of point mutations included 551 NSCLCs; this group of tumors was intentionally composed of both NSCLCs lacking alterations in *EGFR*, *ERBB2 (HER2)*, *ALK*, *ROS1*, *RET*, *NTRK1/2/3*, *MET*, *KRAS*, *NRAS* or *BRAF* genes (*n* = 367) and carcinomas carrying activating mutations in *EGFR* or *KRAS* genes (*n* = 184). Clinical characteristics of the patients are given in Table 1.

**Table 1:** NSCLCs for the extended study of genetic alterations in kinase-encoding genes

**Characteristics**	**Fusion study**	**Mutation study**
Total	1,059	551
Sex		
Male	802 (75.7%)	398 (72.2%)
Female	257 (24.3%)	153 (27.8%)
Age		
Range	21–89	26–89
Median	63	63.5
≤ 50 years old	93 (8.8%)	49 (8.9%)
Smoking status		
Yes	405 (38.2%)	147 (26.7%)
No	222 (21%)	78 (14.2%)
No data	432 (40.8%)	326 (59.2%)

## Results

### Selection of tumors for RNA sequencing study

We initially considered 2,994 NSCLC patients who underwent molecular testing for common *EGFR*, *ERBB2* (*HER2*), *ALK*, *ROS1*, *RET*, *NTRK1/2/3*, *MET*, *KRAS*, *NRAS*, and *BRAF* gene alterations from August 2020 to May 2022. Smoking status was available for 2,034/2,994 (67.9%) of these cases. Presence of driver mutation in the above genes was detected in 369/1,017 (36.3%) smokers vs. 595/1,017 (58.5%) non-smokers (*p* < 0.0001), 776/1,953 (39.7%) males vs. 642/1,041 (61.7%) females (*p* < 0.0001), and 1,237/2,650 (46.7%) patients aged above 50 years vs. 181/344 (52.6%) subjects of 50 years or younger (*p* = 0.0383). The frequency of druggable events in female non-smokers approached 421/623 (67.6%). We further selected for the study 89 non-smoking mutation-negative patients, who were represented by 76 subjects younger than 50 years (24 females and 52 males) and 13 women aged 58–79 years. These tumors were analyzed for alterations in 650 kinase genes.

### Gene fusions

RNA sequencing revealed 93 chimeric transcripts in 89 tumors, with 0–6 fusions per tumor sample. There were 31 translocations involving two chromosomes each and 62 intrachromosomal rearrangements (17 inversions, 27 deletions, and 18 duplications). Thirty-two rearrangements were in-frame, i.e., they were able to produce a potentially functional transcript, while the remaining 61 fusions were out-of-frame. Nine rearrangements occurred more than once in this data set, however, they all were out-of-frame. Tyrosine kinase domain was fully preserved in 9 out of 32 in-frame rearrangements (Table 2), and a part of the kinase portion of the gene was retained in 8 out of these 32 tumors (Table S5). These 17 rearrangements were confirmed by variant-specific PCR and further analyzed in 1,059 NSCLCs, which lacked common alterations in *EGFR*, *ERBB2* (*HER2)*, *ALK*, *ROS1*, *RET*, *NTRK1/2/3*, *MET*, *KRAS, NRAS*, and *BRAF* genes. These efforts led to the identification of two additional tumors with *ADK*::*KAT6B* rearrangement and one carcinoma carrying *RPS6KB1*::*VMP1* fusion. Both these fusions retain only a relatively small portion of the kinase domain of the involved gene, so they are unlikely to render a direct kinase-activating effect. Furthermore, the oncogenic role of *RPS6KB1*::*VMP1* fusion is believed to be attributed not to RPS6KB1 kinase but to the altered function of VMP1 protein. In addition, the recently reported *CLIP1*::*LTK* [16] gene fusion was analyzed in 2,754 NSCLCs, which were negative for all known actionable mutations, however, no new instances of this translocation have been observed.

**Table 2:** Kinase fusions with preserved kinase domain

**Kinase domain**	**Fusion’s name**	**Rearrangement (GRCh38)**	**5’-partner’s function**	**3’-partner’s function**
5’-partner	*MAPK10::LOC107986215*	Intrachromosomal [chr4:3.95Mb]; chr4:86064266:– chr4:81915358:–	Serine/threonine-protein kinase; Neuronal proliferation, differentiation, migration, and programmed cell death	Non-coding RNA
	*STK38::CDC73*	Interchromosomal [chr6--chr1]; chr6:3649671:– chr1:193135391:+	Serine/threonine-protein kinase, transferase; Cell cycle and apoptosis, negative regulator of MAP3K1/2 signaling	Tumor suppressor; Cell cycle, regulation of transcription, Wnt signaling pathway
	*BCR::PKHD1*	Interchromosomal [chr22--chr6]; chr22:23273774:+ chr6:51960026:–	Serine/threonine-protein kinase, transferase; Guanine-nucleotide releasing factor	Receptor; Adhesion, cell motility
	*CDC42BPG::ATG2A*	Intrachromosomal [chr11:0.05Mb]; chr11:64832428:– chr11:64906815:–	Serine/threonine-protein kinase, transferase; Cytoskeletal reorganization, cell invasion	Transporter; Autophagy, lipid transport
	*GALK2::FGF7*	Intrachromosomal [chr15:0.06Mb]; chr15:49328130:+ chr15:49483151:+	Kinase, transferase; Metabolic protein	Growth factor; Heparin-binding, embryonic development, cell proliferation, and differentiation
3’-partner	*CLTC::RPS6KB1*	Intrachromosomal [chr17:0.20Mb]; chr17:59677188:+ chr17:59910562:+	Transporter; Autophagy, cell cycle, cell division, mitosis	Serine/threonine-kinase, transferase; Apoptosis, cell cycle, and regulation of translation
	*ATXN2L::SMG1*	Intrachromosomal [chr16:9.90Mb]; chr16:28825402:+ chr16:18876393:–	Plasma protein; Neurodegenerative disorders	Serine/threonine-kinase; DNA damage, DNA repair, nonsense-mediated mRNA decay
	*WASF2::FGR*	Intrachromosomal [chr1:0.12Mb]; chr1:27414833:– chr1:27617296:–	Actin-binding protein; Changes in cell shape, motility, or function	Non-receptor tyrosine-kinase; Immunity

Chr: chromosome

### Mutations in protein kinase genes

RNA sequencing revealed 601 non-synonymous somatic mutations. We further considered all mutations, which were located within the kinase domain of the involved genes and had combined annotation dependent depletion (CADD) score above 25 (Table 3). None of these 53 substitutions occurred more than once in 89 NGS-analyzed NSCLCs (Table S5). We further tested all these mutations in 367 NSCLCs lacking driver mutations and 184 carcinomas carrying activating mutations in *EGFR* or *KRAS* genes. No new instances of these mutations were detected.

**Table 3:** Mutations in kinase domains

**Chr**	**Position**	**REF**	**ALT**	**Gene**	**Protein**	**Effect**	**CADD**
1	59321726	G	T	*FGGY*	p.Trp59Cys	missense_variant	29.7
1	64177651	G	T	*ROR1*	p.Cys537Phe	missense_variant	25.6
7	151057136	G	A	*CDK5*	p.Ala21Val	missense_variant	29.5
4	86101117	G	A	*MAPK10*	p.Pro222Leu	missense_variant	27.8
6	7402834	G	A	*RIOK1*	p.Cys235Tyr	missense_variant	31
1	162500036	G	T	*UHMK1*	p.Ser117Ile	missense_variant	28.5
3	142496516	C	G	*ATR*	p.Asp1915His	missense_variant	25.4
17	28122673	G	T	*NLK*	p.Val177Phe	missense_variant	27.9
19	40238013	G	C	*AKT2*	p.Leu263Val	missense_variant	25.1
6	115942559	C	G	*FRK*	p.Cys458Ser	missense_variant	25.3
9	127789200	A	G	*CDK9*	p.Tyr259Cys	missense_variant	28.5
5	56883589	G	C	*MAP3K1*	p.Trp1243Cys	missense_variant	32
19	18123365	G	T	*MAST3*	p.Arg183Leu	missense_variant	26.4
1	204440257	C	A	*PIK3C2B*	p.Trp1105Leu	missense_variant	29.1
10	103999254	G	T	*SLK*	p.Met241Ile	missense_variant	26
2	147918462	T	A	*ACVR2A*	p.Phe278Ile	missense_variant	25.1
2	172564574	T	A	*PDK1*	p.Ile181Asn	missense_variant	25.8
1	179117461	C	T	*ABL*	p.Asp427Asn	missense_variant	28.4
1	205528150	C	G	*CDK18*	p.Ser349Cys	missense_variant	28.8
7	100807468	C	A	*EPHB4*	p.Arg744Leu	missense_variant	29.4
6	10803770	C	G	*MAK*	p.Glu205Gln	missense_variant	25.3
16	23695909	CС	AA	*ERN2*	p.Gly532Leu	missense_variant	25.4
7	137645473	T	A	*DGKI*	p.Lys268Met	missense_variant	34
8	140746862	T	C	*PTK2*	c.1820-2 T > C	splice_acceptor_variant	33
16	18850461	C	A	*SMG1*	p.Asp1687Tyr	missense_variant	27.5
7	140673943	C	A	*ADCK2*	p.Pro205Thr	missense_variant	25.6
13	26337623	C	T	*CDK8*	p.Ser62Leu	missense_variant	29.9
15	98957103	G	T	*IGF1R*	p.Met1255Ile	missense_variant	29.1
18	21045409	C	A	*ROCK1*	p.Gly158Val	missense_variant	25.8
4	106250454	C	A	*TBCK*	p.Asp208Tyr	missense_variant	25.5
3	38397157	A	G	*XYLB*	p.His452Arg	missense_variant	25.2
11	67434052	G	A	*RPS6KB2*	p.Val322Met	missense_variant	26.5
2	200853964	A	G	*CLK1*	p.Leu459Ser	missense_variant	27.3
21	44325220	G	T	*PFKL*	p.Gly649Cys	missense_variant	26.7
4	78844976	T	C	*BMP2K*	p.Phe199Leu	missense_variant	28.1
8	27828135	C	T	*PBK*	p.Gly41Asp	missense_variant	25.7
9	4719192	C	A	*AK3*	p.Trp129Cys	missense_variant	32
1	32275576	T	G	*LCK*	p.Phe129Val	missense_variant	31
22	20729351	C	A	*PI4KA*	p.Trp1548Cys	missense_variant	28.5
9	99144879	G	A	*TGFBR1*	p.Gly378Glu	missense_variant	29.4
3	142493161	C	T	*ATR*	p.Glu2017Lys	missense_variant	29.2
15	50574399	C	G	*TRPM7*	p.Arg1728Thr	missense_variant	25.6
2	241499292	G	A	*STK25*	p.Pro184Ser	missense_variant	28.4
1	46023823	G	C	*MAST2*	p.Lys541Asn	missense_variant	26.5
9	130854948	G	T	*ABL*	p.Arg153Leu	missense_variant	29
7	151077742	G	T	*FASTK*	p.Leu360Met	missense_variant	25.5
19	45778550	T	C	*DMPK*	p.Tyr175Cys	missense_variant	29.8
19	17835206	C	A	*JAK3*	p.Gly642Cys	missense_variant	26.6
18	42057984	G	T	*PIK3C3*	p.Gly789Cys	missense_variant	31
2	219481341	C	G	*SPEG*	p.Arg1803Gly	missense_variant	26
19	1220692	G	T	*STK11*	p.Asp237Tyr	missense_variant	32
5	178612457	A	G	*CLK4*	p.Leu337Ser	missense_variant	29.4
11	67433445	A	C	*RPS6KB2*	p.Lys302Gln	missense_variant	25.5

Chr: chromosome; REF: reference allele; ALT: alternative allele; CADD: combined annotation dependent depletion

### Expression analysis

**Figure 1:**
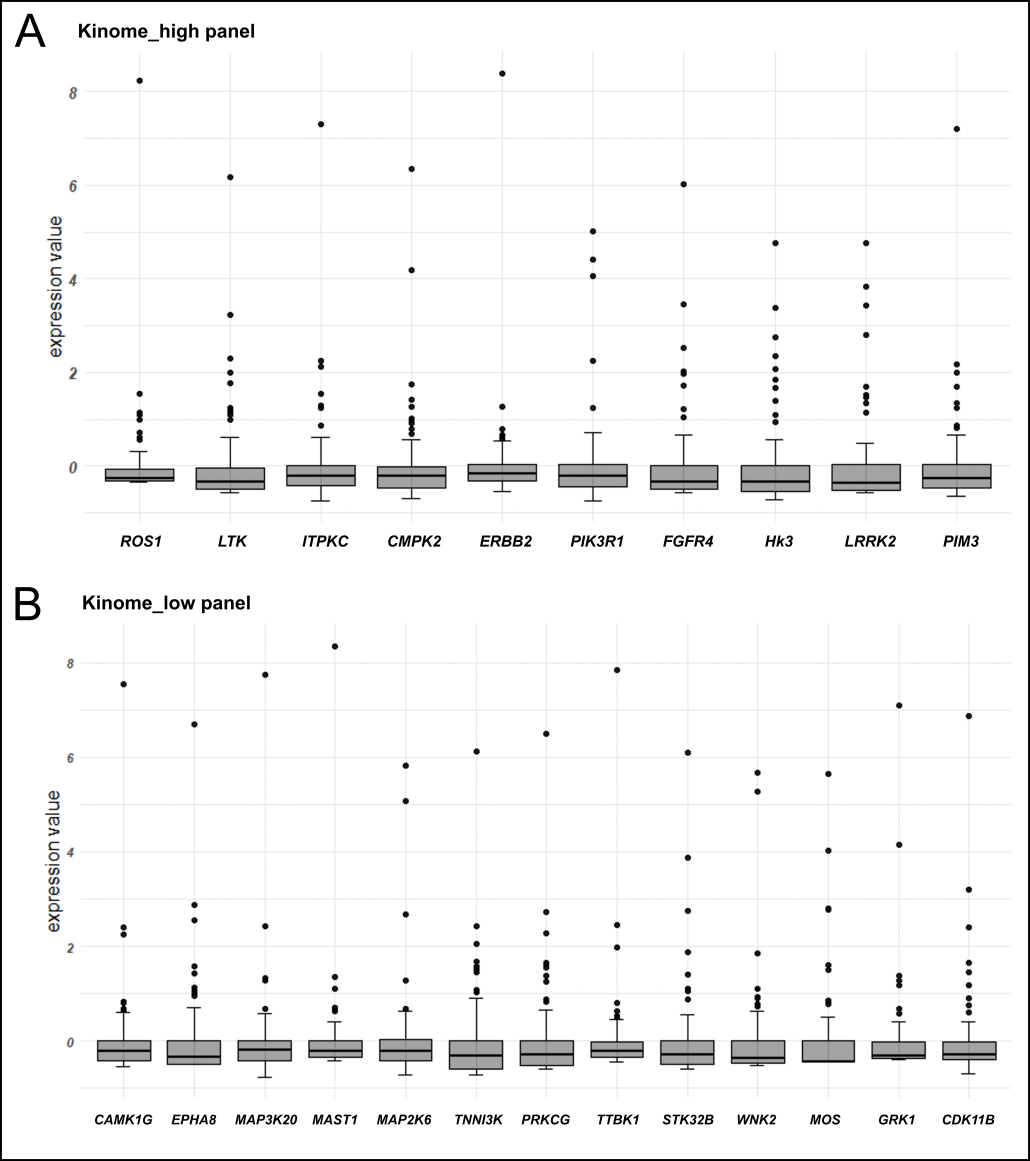
**Graphical demonstration of instances of kinase gene overexpression.** (**A**) The analysis of genes with high level of average expression, in which a 100-fold increase of RNA transcription as compared to the 75%-quartile value is taken as a threshold; (**B**) the analysis of genes with low/medium level of average expression, in which a 250-fold increase of RNA transcription as compared to the 75%-quartile value is taken as a threshold

Instances of kinase overexpression were observed in 20/89 (22.5%) NSCLCs; a single kinase gene was activated in 14 tumors, while six NSCLCs demonstrated overexpression of more than one kinase (Table S5). PIK3R1 transcription was significantly elevated in 3/89 (3.4%) tumors; CMPK2, MAP2K6, WNK2, and GRK1 kinases were overexpressed in 2 tumors each; single instances of gene overexpression were observed for 18 kinases (Figure 1). Overall, 7/89 (7.9%) NSCLCs had overexpressed kinases amenable to therapy with clinically available compounds (Table 4).

**Table 4:** Overexpressed kinases and clinically available therapy

**Gene**	**Number of cases**	**Therapy [reference]**
*ROS1*	1	Crizotinib, entrectinib [17, 18]
*LTK*	1	Lorlatinib [16]
*ITPKC*	1	No
*CMPK2*	2	No
*ERBB2*	1	Trastuzumab and other HER2 inhibitors [19, 20]
*PIK3R1*	3	Binimetinib [21]
*FGFR4*	1	Futibatinib, erdafitinib, [22, 23]
*HK3*	1	No
*LRRK2*	1	No
*PIM3*	1	No
*CAMK1G*	1	No
*EPHA8*	1	No
*MAP3K20 (ZAK)*	1	No
*MAST1*	1	No
*MAP2K6*	2	No
*TNNI3K*	1	No
*PRKCG*	1	No
*TTBK1*	1	No
*STK32B*	1	No
*WNK2*	2	No
*MOS*	1	No
*GRK1*	2	No
*CDK11B*	1	No

## Discussion

This study indicates that the majority of driver mutations in kinase genes, which are relevant to NSCLC pathogenesis, have already been identified, and no new major contributors are expected to emerge in the future. Consequently, the concept of personalization of NSCLC therapy based on the mutation-tailored selection of kinase inhibitors has its limits, even for young-onset and/or non-smoking and/or female patients. Even presumably recurrent mutations, which have been identified recently, have vanishingly low frequency. For example, our study failed to detect potentially druggable *CLIP1*-*LTK* rearrangements in a large series of NSCLCs, although the initial report of Izumi et al. [16] suggested its frequency to be around 0.4%.

Some of the identified kinase gene rearrangements have been reported in prior studies. For example, *ADK*::*KAT6B* fusion (NSCLC P13756) was observed in a woman with ovarian cancer [24]. *RPS6KB1*::*VMP1* (NSCLC P13041) translocation is a recurrent event in esophageal cancer [25]. Several tumors with *WASF2*::*FGR* rearrangements (NSCLC P19401) are described in the TCGA database [26]. *EGFR*::*SEPTIN14* (NSCLC P22152) has been described in a NSCLC patient responding to icotinib as well as an acquired osimertinib resistance mutation in a patient with *EGFR* exon 19 deletion [27, 28].

Our study demonstrates some promise of systematic analysis of overexpressed drug targets in mutation-negative tumors. The exclusion of NSCLCs with known driver mutations appears to be essential in this context: for example, a clinical trial involving HER2 tumor targeting demonstrated the advantage of this therapy only in patients with *KRAS* mutation-negative tumors, while patients with RAS activation had no clear benefit from the treatment [29]. Still, the limitations of the present study must be taken into account. Although we selected a conservative threshold for discriminating between kinase-overexpressing and non-overexpressing tumors, it is essential to recognize that RNA sequencing is more error-prone in the analysis of gene expression as compared to methods utilizing more direct measurement of the transcript levels. For example, one of the analyzed tumors (NSCLC P21113) apparently contained 6 overexpressed kinase genes, which may be attributed to some features of bioinformatic normalization analysis rather than to biological factors. It appears that only activated members of the MAPK signaling cascade are reliable therapeutic targets. Indeed, interference with other signaling kinases, for example, members of phosphatidylinositol 3-kinases, demonstrated limited efficacy in an agnostic setting [30].

It is necessary to acknowledge that the analysis of frequencies of the newly identified kinase gene alterations involved NSCLCs with somewhat distinct clinical characteristics as compared to the discovery cohort. Indeed, the kinome NGS analysis was performed in a highly selected group of patients who did not have actionable gene alterations despite being young and/or female non-smokers. This approach is rational because young age, female gender, and non-smoking status are strongly associated with a high frequency of druggable genetic lesions, particularly kinase gene fusions [12]. Given that all 89 tumors from the discovery cohort were negative for *EGFR*, *ERBB2 (HER2)*, *ALK*, *ROS1*, *RET*, *NTRK1/2/3*, *MET*, *KRAS*, *NRAS*, or *BRAF* alterations, and knowing that activating events in the MAPK cascade are mutually exclusive [31], it appears likely that these patients were enriched by yet unknown, potentially druggable mutations. However, it is virtually impossible to collect a similar group of patients for the validation study, because of the low incidence of lung cancer in young non-smokers and high frequency of *MAPK* gene alterations in this category of NSCLC. Consequently, patients included in the validation cohort had a higher median age and male-to-female ratio, and, in contrast to the discovery cohort, contained a significant number of smokers and subjects with an unknown history of tobacco use. Nevertheless, this limitation is compensated by a sufficient sample size of the validation study, therefore, it is unlikely that truly recurrent events were missed in our data set.

In conclusion, a systematic study of kinase genes in non-smoking young-onset and/or female patients with no known mutations in members of the MAPK signaling cascade failed to identify novel frequent genetic alterations. Instances of apparent overexpression of *ROS1*, *LTK*, *FGFR4*, and MAPK family genes deserve further investigation.

## Abbreviations

CADD: combined annotation dependent depletion
FFPE: formalin-fixed paraffin-embedded
NGS: next generation sequencing
NSCLCs: non-small cell lung carcinomas

## Supplementary materials

The supplementary tables for this article are available at: https://www.explorationpub.com/uploads/Article/file/1002330_sup_1.xlsx.

## Declarations

### Availability of data and materials

The raw data supporting the conclusions of this manuscript will be made available by the authors, without undue reservation, to any qualified researcher.

### Copyright

© The Author(s) 2025.
